# Polymer grafted magnetic graphene oxide as a potential nanocarrier for pH-responsive delivery of sparingly soluble quercetin against breast cancer cells

**DOI:** 10.1039/d1ra05382e

**Published:** 2022-01-19

**Authors:** Monika Matiyani, Anita Rana, Mintu Pal, Sravendra Rana, Anand B. Melkani, Nanda Gopal Sahoo

**Affiliations:** Prof. Rajendra Singh Nanoscience and Nanotechnology Centre, Department of Chemistry, D. S. B. Campus, Kumaun University Nainital-263001 Uttarakhand India ngsahoo@yahoo.co.in; Department of Pharmacology, AIIMS Bathinda Punjab India; University of Petroleum & Energy Studies (UPES), School of Engineering, Department of Chemistry, Energy Acres Bidholi Dehradun 248007 India

## Abstract

In this work, polymer grafted magnetic graphene oxide (GO–PVP–Fe_3_O_4_) was successfully synthesized for efficient delivery of anticancer drug. Firstly, GO was functionalized with the hydrophilic and biocompatible polymer polyvinylpyrrolidone (PVP) and then grafted with magnetic nanoparticles (Fe_3_O_4_) through an easy and effective chemical co-precipitation method. Quercetin (QSR) as an anticancer drug was loaded onto the surface of GO–PVP–Fe_3_O_4_*via* non-covalent interactions. The drug loading capacity was as high as 1.69 mg mg^−1^ and the synthesized magnetic nanocarrier shows pH-responsive controlled release of QSR. The cellular cytotoxicity of the synthesized nanocarrier with and without drugs was investigated in human breast cancer MDA MB 231 cells and their effects compared on non-tumorigenic epithelial HEK 293T cells. These results reveal that the drug loaded GO–PVP–Fe_3_O_4_ nanohybrid was found to be more toxic than the free drug towards MDA MB 231 cells and exhibits biocompatibility towards HEK 293T cells. Overall, a smart drug delivery system including polymer grafted magnetic graphene oxide as a pH-responsive potential nanocarrier could be beneficial for targeted drug delivery, controlled by an external magnetic field as an advancement in chemotherapy against cancer.

## Abstract 2

Polymer grafted magnetic graphene oxide (GO–PVP–Fe_3_O_4_) as an efficient nanocarrier for the delivery of anticancer drug quercetin.

## Graphical abstract

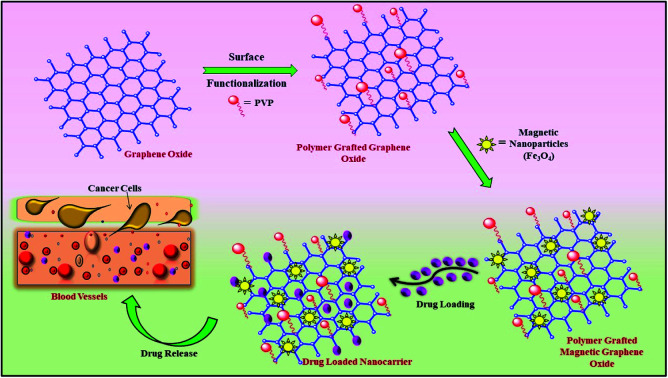

## 1. Introduction

Cancer is the one of the most devastating diseases to human health and a leading cause of death worldwide. In the past few years, a number of chemotherapeutic drugs such as doxorubicin (DOX), curcumin, quercetin (QSR), camptothecin, *etc.* have been widely employed for the treatment of various kinds of cancer. Besides having numerous advantages, these drugs also exhibit a number of serious drawbacks such as non-specific distribution, inadequate drug concentrations in cancerous cells, overdose, short distribution period and low bioavailability as well as severe toxicity to healthy cells.^1,2^ Furthermore, it is well known that most of the anticancer drugs, due to their hydrophobic nature, are insoluble or sparingly soluble in aqueous and physiological solution. Particularly, a well-known drug quercetin (QSR), 3,3′,4′,5′,7-pentahydroxyflavone belongs to the class of flavonoids that possess anticancer activity as well as a wide variety of pharmacological and biological properties.^3–5^ However, QSR has suffer from low bioavailability due to its limited aqueous solubility which limits its biological applications.^6^ To suppress the above limitation of the drug, there is a need for an effective drug carrier which can increase the solubility of the drug in aqueous and physiological media as well as enhance drug efficacy and lower drug toxicity due to controlled drug release. In this regard, nanocarriers have been emerged as promising tools having excellent ability to deliver the drug at respective sites due to their nanosized structure and large surface area. There are a number of nanocarriers including dendrimers, liposomes, polymeric micelles, carbon nanomaterials, metal oxide nanoparticles, *etc.* which can effectively deliver the drug to the cancerous or tumor cells and show remarkable potential in the field of drug delivery.

Graphene, a two-dimensional mono-layer sheet of sp^2^ hybridized carbon atoms, in which carbon atoms are arranged in honeycomb-like structure. Graphene has gained remarkable attention in the past few decades due to its extraordinary properties such as great mechanical strength, excellent chemical stability, large surface area and *etc.*^7–10^ Among various derivatives of graphene, graphene oxide (GO) has been extensively studied as one of the most promising drug carriers for effective drug delivery due to its distinctive chemical and physical characteristics. The presence of abundant hydrophilic oxidative functional groups on the surface of GO are responsible for its better dispersibility in water as well as in physiological solution. In addition, these oxygenated groups are capable to form covalent or non-covalent bonds with other functional moieties in order to modify the surface of GO.^11–13^ GO has also shown excellent ability to load chemotherapeutic drugs and other chemical moieties through hydrogen bonding, electrostatic interactions and π–π interactions.^14,15^ Despite these, GO also suffer from its poor biocompatibility and exhibit lack of stability in physiological solution which restricts its application in drug delivery. To overcome these limitations, the surface modification of GO is enormously gaining interest in improving the potential of GO.^16^ The functionalized GO can also extend hydrophilicity to hydrophobic drugs and can be used as the efficient and effective nanocarrier for anticancer drug.^17^

In recent years, different hydrophilic and biocompatible polymers^18–23^ have been used for the functionalization of GO which increases its biocompatibility, solubility and stability in biological solutions as well as improve the dissolution of water-insoluble anticancer drugs. However, to enhance therapeutic efficiency of anticancer drug and induces targeting ability, GO-based nanocarriers are incorporate with the magnetic nanoparticles.^24–27^ In magnetic nanoparticles, Fe_3_O_4_ nanoparticles have received much attention and also, most commonly used as a source of magnetic nanoparticles due to its unique features such as good biocompatibility, ease of preparation, high superparamagnetic character, low toxicity, excellent chemical stability.^28,29^ It is well known fact that GO and Fe_3_O_4_ nanoparticles are showed the agglomeration separately in the solution media. But, when these are come together, they showed the synergic effect towards each other, magnetic nanoparticles avoid the lapping of GO sheets and on the other hand, GO avoid the agglomeration of magnetic nanoparticles.^30^ Yang *et al.* synthesized GO–Fe_3_O_4_ based nanocarrier for the loading of DOX and reported 1.08 mg mg^−1^ DOX loading capacity.^27^ On the other hand, Tiwari *et al.* prepared GO–PVP based nanocarrier for dual delivery of Gefitinib (GEF) and QSR. They found that the loading capacity of GO–PVP for GEF and QSR (single drug systems) are 20% and 14%, respectively, and GO–PVP–QSR–GEF (dual drug system) shows loading capacity of GEF and QSR are 46% and 20%, respectively.^21^

In the present work, we have successfully synthesized polyvinylpyrrolidone-grafted magnetic GO (GO–PVP–Fe_3_O_4_) as a nanocarrier for the loading and delivery of anticancer drug quercetin (QSR). The prepared nanocarrier shows the excellent loading capacity for the QSR of 1.69 mg mg^−1^ which is higher in compared to the previously reported work in GO-based nanocarrier for several anticancer drugs. Also, it exhibits the pH-triggered release behavior for QSR. Further, QSR loaded GO–PVP–Fe_3_O_4_ nanocarrier has been investigated for cytotoxicity against non-tumorigenic HEK 293T and human breast cancer MDA MB 231 cells. QSR loaded GO–PVP–Fe_3_O_4_ nanocarrier exhibits more cytotoxic effects towards the MDA MB 231 breast cancer cell in comparison to the pure QSR; whereas no significant cytotoxic effects in the normal cells. Our novel findings reveal that the synthesized magnetic nanocarrier could be a promising candidate for cancer therapy.

## 2. Experimental

### 2.1 Materials

Expanded graphite powder (average particle size ∼100 μm) and quercetin (QSR) were purchased from Sigma-Aldrich. Polyvinylpyrrolidone (PVP, *M*_w_ ∼ 40 000) was obtained from Calbiochem. Hydrogen peroxide (H_2_O_2_, 30 wt% aqueous solution), sodium nitrate (NaNO_3_), sulphuric acid (H_2_SO_4_), potassium permanganate (KMnO_4_), iron(ii) sulfate heptahydrate (FeSO_4_·7H_2_O), iron(iii) chloride hexahydrate (FeCl_3_·6H_2_O), sodium hydroxide (NaOH), fetal bovine serum (FBS), penicillin–streptomycin, sulforhodamine B (SRB) assay and Eagle's minimum essential medium (MEM) with NEAA were obtained from Himedia. *N*,*N*′-dicyclohexyl carbodiimide (DCC) and 4-dimethylaminopyridine (DMAP) were obtained from Sisco Research Laboratories Pvt. Ltd (SRL).

### 2.2 Preparation of GO

GO was synthesized from expanded graphite powder using a modified Hummer's method.^31^ Briefly, 1 g of expanded graphite powder was mixed with 1 g of NaNO_3_ and 46 mL of H_2_SO_4_ and stirred continuously for half an hour at 27 °C. After that, under ice-water bath condition, 6 g of KMnO_4_ was added slowly to the obtained slurry mixture with stirring followed by heating at 32 °C for 2 hours. After vigorously stirring for 2 hours, 92 mL double distilled water (DD water) was added slowly to the solution mixture followed by maintaining the temperature at 95 °C with stirring over about 1 hour. The resultant mixture was further stirred for 2 hours after adding 200 mL of DD water. Later on, 30 mL of H_2_O_2_ (30 wt% aq.) was added to the mixture and continuously stirred at room temperature for 1 hour. Lastly, the oxidized product was purified with 10% HCl solution and repeatedly washing with DD water until the final product has been neutralized.

### 2.3 Preparation of PVP grafted GO (GO–PVP)

**Scheme 1:**
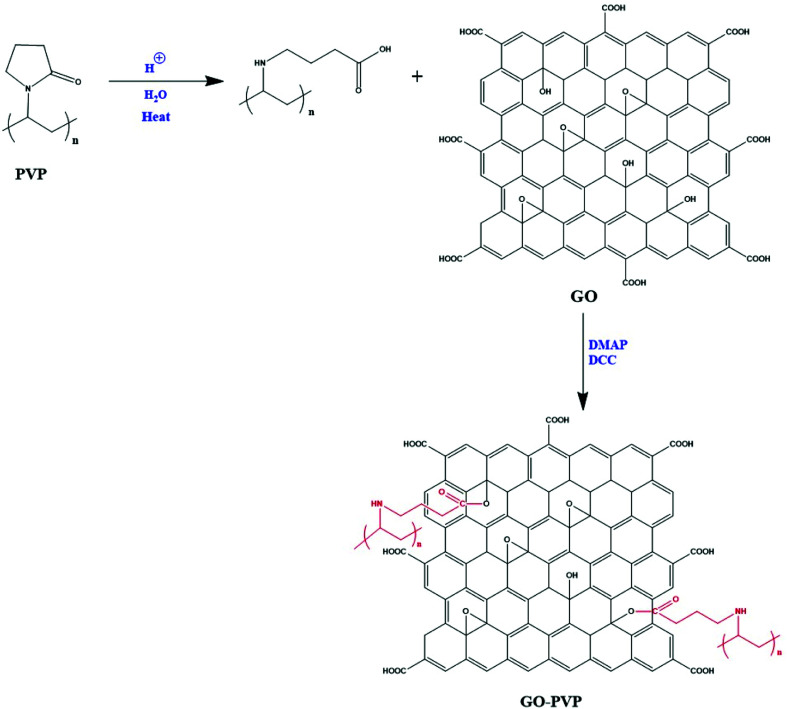
Detailed mechanism of coupling between GO and PVP.

GO was functionalized with PVP through carbodiimide activated esterification method.^32,33^ In this method, firstly, GO solution was prepared by dissolving 50 mg of GO in 15 mL of DMSO and then the solution was sonicated for 45 min to obtain a brown-colored homogeneous solution. After that, a definite quantity of PVP heated for 15 min with small amount of dilute hydrochloric acid. Further, an appropriate quantity of catalysts DCC and DMAP were added gradually to the solution of PVP and continuously stirred for 30 min. Then, 15 mL of GO solution was added to the resulting mixture followed by stirring for three days at a heating temperature of 50 °C. After three days continuous stirring, the solution was filtered through a PTFE microporous membrane (0.2 μm) and the resulting solid was washed with acetone and dimethylformamide (DMF). Then, the solid was dissolved in hot water followed by the filtration through 0.2 μm nylon membrane to remove unreacted PVP. Finally, the filtered residue was collected after washing with a significant amount of hot water and dried in a vacuum oven. The detailed mechanism of coupling between GO and PVP is shown in Scheme 1.

### 2.4 Preparation of Fe_3_O_4_ magnetic nanoparticles functionalized GO–PVP (GO–PVP–Fe_3_O_4_)

**Scheme 2:**
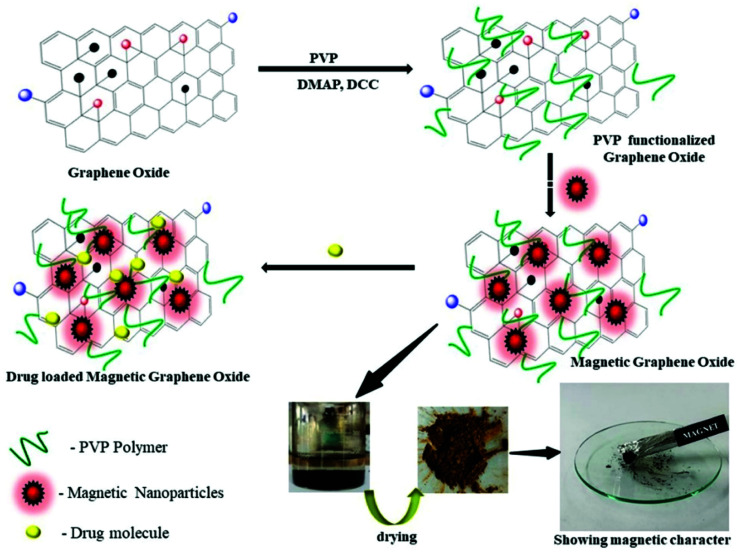
Schematic representation of synthesis of magnetic graphene oxide and drug loading on magnetic graphene oxide.

The functionalization of GO–PVP with Fe_3_O_4_ magnetic nanoparticles has been done by chemical co-precipitation method (Scheme 2).^34^ In a typical procedure, 0.5 g of GO–PVP dispersed in 50 mL of DD water and sonicated for 30 min. The 100 mL solution containing 556 mg of FeSO_4_·7H_2_O and 1081 mg of FeCl_3_·6H_2_O salts continuously stirred at 40 °C for about 30 min and thereafter reaction mixture was adjusted to pH 4 by the addition of 1 M NaOH solution. The next step was the addition of sonicated GO–PVP solution to the mixture of Fe(ii)/Fe(iii) followed by the stirring for 1 hour. Finally, 1 M NaOH solution was added to the resulting solution to reach pH 10 and then the solution was allowed to stand for 1 hour. Then, the resultant precipitate was separated with the help of magnet and repeatedly washed with DD water and subsequently, dried overnight in an oven at 50 °C.

### 2.5 Loading of QSR on GO–PVP–Fe_3_O_4_ composite

Briefly, 4 mL of 1 mg mL^−1^ GO–PVP–Fe_3_O_4_ composite in phosphate buffer solution (PBS, pH 7.4) was mixed with 2 mL of 4 mg mL^−1^ QSR solution in DMSO and then stirred for 24 hours at room temperature. The drug-loaded nanocarrier was then magnetically separated and washed 2–3 times with DD water. Finally, the purified drug-loaded nanocarrier dried in a vacuum oven at 40 °C.

### 2.6 *In vitro* drug release

To study drug release behavior, 10 mg of QSR loaded GO–PVP–Fe_3_O_4_ nanocarrier was added in a beaker and dispersed by adding 20 mL of pH 4 and 7.4 buffer solution separately. Then, beakers were placed in a shaker constantly for 3 days at room temperature. At definite time intervals, 3 mL supernatant solution was taken out from the beaker and replaced by equal volume of fresh buffer solution to keep a constant amount of solution in a beaker during the whole experiment. Finally, the collected supernatants were examined by UV-visible spectrophotometer to determine the amount of the drug released.

### 2.7 Fabrication of QSR, GO–PVP–Fe_3_O_4_ and GO–PVP–Fe_3_O_4_–QSR nanocomposite-modified electrode

The electrodes were prepared by drop-casting method as reported previously.^35^ Briefly, 1 mg of QSR, GO–PVP–Fe_3_O_4_ and GO–PVP–Fe_3_O_4_–QSR were dispersed in 1 mL of DD water separately and sonicate for 1 hour to make a homogenous mixture. For the preparation of electrode, initially glassy carbon electrode (GCE) was polished with the alumina power, then 60 μL of each QSR, GO–PVP–Fe_3_O_4_ and GO–PVP–Fe_3_O_4_–QSR were drop cast separately onto the surface of GCE and dried at 60 °C in the oven. After that, the prepared electrodes were immersed in 0.1 M PBS (pH 7.4) for electrochemical measurements in three electrode system with Pt wire and Ag/AgCl as the counter and reference electrodes, respectively. This study was done for the confirmation of QSR loading onto the GO–PVP–Fe_3_O_4_.

### 2.8 Cell culture and *in vitro* cytotoxicity assay

The human breast cancer MDA MB 231 cell line and non-tumorigenic HEK 293T epithelial cell line were obtained from the National Center for Cell Sciences (NCCS), Pune, India. The cells were cultured and maintained by using a suitable culture medium containing 1% penicillin/streptomycin as an antibiotic and 10% fetal bovine serum (FBS) in a 5% CO_2_ incubator at 37 °C in a humidified environment. As reported previously,^36^ sulforhodamine B (SRB) assay was carried out to evaluate the relative cell survival percentage. Briefly, cells at a concentration of 3000 cells per well were seeded in 96 well microtiter plates and treated with various concentrations (0, 10, 20, 30, and 50 μg mL^−1^) of the synthesized nanocarriers (with and without drug). After the treatment of 48 hours, each well was filled with 25 μL of cold 50% trichloroacetic acid (TCA) and the plates were incubated for 1 hour at 4 °C. To eliminate the serum proteins and TCA solution, the wells were rinsed with water and then, the plates were dried. Then, each well was filled with 50 μL of a 0.4% SRB solution and the plates were incubated at room temperature for 30 min, after which the plates were washed with 1% acetic acid to remove excess dye. The plates were air dried and a light microscope was used to capture the images. For quantitative measurements, 100 μL of 10 mM Tris base solution was added to each well to solubilized protein bound dye and the absorbance was measured at 565 nm. The cytotoxicity of drug-loaded nanocarriers towards MDA MB 231 and HEK 293T cell lines were evaluated by IC_50_ (half maximal inhibitory concentration) value.

## 3. Characterization

The synthesized nanocarrier and its related systems were confirmed by some advanced characterizing techniques such as Raman (Research India, RIRMLP1519), Fourier transform infrared (FT-IR), field emission scanning electron microscopy (FESEM). Also, ImageJ software was used for morphological illustration of prepared materials. Thermogravimetric analysis (TGA) was performed from 30 °C to 600 °C at a heating rate of 10 °C min^−1^ under N_2_ atmosphere by using Thermo Gravimetric Analyzer TGA 4000, PerkinElmer. Thereafter, the attachment of drug over the synthesized nanocarrier was also confirm by the electrochemical study using Electrochemical workstation, Metrohm Autolab. The drug loading capacity, entrapment efficiency and *in vitro* drug release behavior were studied using by UV-vis spectroscopy. The biocompatibility and cellular toxicity of synthesized nanocarriers were investigated by using SRB assay.

## 4. Results and discussion

### 4.1 Raman spectra analysis

**Fig. 1:**
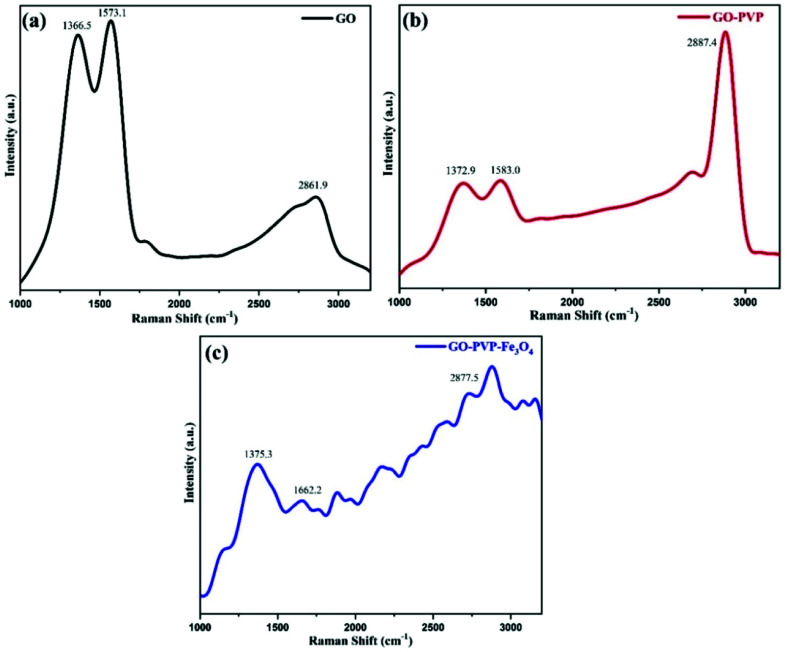
Raman spectra of (a) GO (b) GO–PVP and (c) GO–PVP–Fe_3_O_4_.

Raman spectroscopy is a non-destructive and precise analysis technique to identify and characterized different types of carbon materials and also provides information about the structural variations during chemical processes. The Fig. 1 shows the Raman spectra of GO, GO–PVP and GO–PVP–Fe_3_O_4_. The Raman spectra of GO exhibit two prominent peaks at 1366.5 cm^−1^ and 1573.1 cm^−1^. The peak at 1366.5 cm^−1^ assign to the D-band or deformation band of GO which is due to the stretching vibration of sp^3^ C-atoms resulting from the conversion of sp^2^ C-atoms of graphite to sp^3^ C-atoms of GO during the oxidation of graphite. The peak at 1573.1 cm^−1^ represents the G-band of GO which is originating from the vibrations of sp^2^ C-atoms. In addition to D and G bands of GO, the Fig. 1(a) shows the 2D band at 2861.9 cm^−1^ which represents purity, formation of graphene structure and number of layers present in GO.^37^ The intensity ratio of 2D and G band *i.e.*, *I*_2D_/*I*_G_ shows the number of sheets in GO. According to the Raman spectra of GO, the *I*_2D_/*I*_G_ ratio is 0.48, representing the existence of more than two layers of GO.

The Raman spectra of GO–PVP also exhibit peaks at 1372.9 cm^−1^, 1583.0 cm^−1^ and 2887.4 cm^−1^ which are respectively assign to D-band, G-band and 2D-band (Fig. 1(b)). The intensity ratio of two characteristic band D and G *i.e.*, *I*_D_/*I*_G_ ratio, determines the degree of disorder present in graphene sheet. The *I*_D_/*I*_G_ ratio for GO and GO–PVP are 0.96 and 0.99 respectively. This increase in the intensity ratio from 0.96 to 0.99, resulting from the transformation of sp^2^ hybridized indicating the successful grafting of PVP carbon to sp^3^ C-atoms after the functionalization of GO with PVP, on GO surface. Fig. 1(c) shows the Raman spectra of GO–PVP–Fe_3_O_4_ with disrupted of D, G and 2D bands and also exhibit slightly increased *I*_D_/*I*_G_ ratio up to 1.10, which may be due to the structural distortions induced by Fe_3_O_4_ nanoparticles. These data confirm the attachment of Fe_3_O_4_ on GO–PVP surface.^38,39^

### 4.2 FTIR spectra analysis

**Fig. 2:**
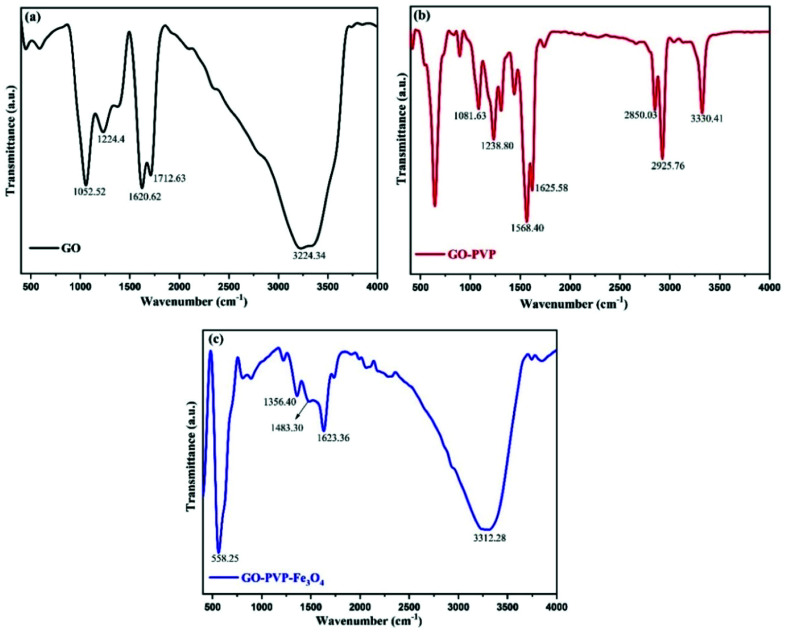
FTIR spectra of (a) GO (b) GO–PVP and (c) GO–PVP–Fe_3_O_4_.

FTIR spectroscopy is a technique to confirms the formation of new bonds and presence of different functional groups in the compound. Within a certain range of wavenumber or frequency, each functional group vibrate with a particular frequency which is depends upon the geometry, relative mass of atom and bonding force constant. Therefore, different chemical functional groups will vibrate at different frequencies. The Fig. 2 shows the FTIR spectra of GO, GO–PVP and GO–PVP–Fe_3_O_4_ nanocomposite which are performed at 400–4000 cm^−1^ wavenumber range. The FT-IR spectra of GO exhibit a broad and intense absorption peak at 3224.34 cm^−1^ which originates from the stretching vibration of O–H. This broad peak is a characteristic peak of hydroxyl (–OH) group. The absorption peaks at 1712.69 cm^−1^ and 1620.62 cm^−1^ are respectively assigned to the stretching vibration of C=O of carboxyl group and aromatic stretching vibration of C=C bond (originating from the graphite domain which was not undergo complete oxidation). Absorptions at 1224.4 cm^−1^ and 1052.52 cm^−1^ are respectively corresponds to the C–O–C (epoxy) and C–O (alkoxy) stretching vibrations. The presence of these absorption peaks indicated that the GO nanosheets containing abundant oxygenated functional groups were synthesized successfully.

In FT-IR spectra of GO–PVP, several new and intense peaks were appeared in addition to the absorption peaks in the spectra of GO due to the functionalization of GO with PVP. The absorption peak at 3330.41 cm^−1^ is corresponds to the stretching vibration of N–H bond of secondary amine group. Additionally, the absorption peak at 2925.76 cm^−1^ and 2850.03 cm^−1^ appeared as a sharp doublet, which were respectively due to the asymmetric and symmetric methylene (–CH_2_–) stretching. The absorption peaks at 1623.36 cm^−1^, 1568.40 cm^−1^ and 1435 cm^−1^ are due to the characteristic stretching vibration of C=C bond, bending vibration of N–H and O–H bond respectively. The FTIR spectra of GO–PVP also exhibit an absorption peak at 1238.80 cm^−1^ which assigned to the C–O stretching vibration of ester functional group which is formed during the functionalization. Generally, the validation of successful functionalization was done with the help of FTIR analysis. In the FTIR spectra of GO–PVP, the broad absorption peak of –OH group which is appeared around 3224 cm^−1^ in case of GO was completely disappeared after the functionalization with PVP. This reflect that the hydroxyl groups of GO may be involved in the formation of new bonds during the functionalization. Also, an additional peak was appeared around 1239 cm^−1^ due to the formation of new C–O bond of ester functional group. Thus, based on the above results, we can conclude that –OH groups of the GO involve in the formation of ester during the functionalization with PVP. This confirms the successful functionalization of GO with PVP.

FT-IR analysis can also confirm the successful attachment of Fe_3_O_4_ nanoparticles onto GO nanosheet by the presence of absorption peak at 558.25 cm^−1^. This peak corresponds to the stretching vibration of Fe–O and represents the formation of the coordination bond (C–O–Fe) between Fe_3_O_4_ nanoparticles and COO^−^ of GO. Moreover, the two new absorption peaks at 1484.3 cm^−1^ and 1356.4 cm^−1^ demonstrate the existence of interaction of Fe_3_O_4_ nanoparticles with hydroxyl and carbonyl groups of GO.^40^

### 4.3 SEM-EDX analysis

**Fig. 3:**
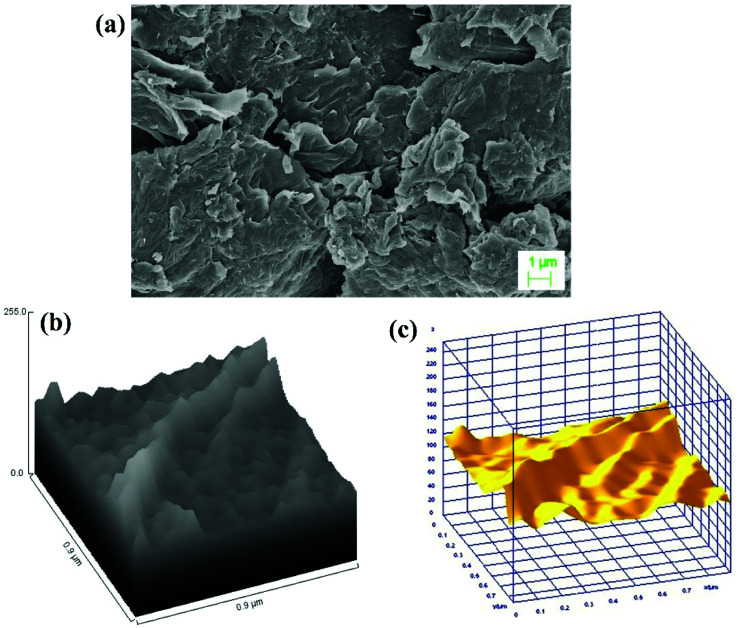
(a) SEM image of GO, (b) Hill stack plot of GO and (c) 3D surface morphology of GO.

The surface morphology and elemental composition of GO, GO–PVP and GO–PVP–Fe_3_O_4_ materials were characterized by SEM-EDX and ImageJ software. The Fig. 3(a) is the SEM image of GO which shows scrambled and sheets like structure. The sheets are look somewhat thicker on the edges which is due to presence of oxygenating groups in GO. The Fig. 3(b) and (c) represent the hill stack profile and 3D surface morphology of GO respectively which shows uneven hills, curvy and thick corner of the sheet as well as suggesting the wrinkled sheet like morphology of GO, shows the good agreements with SEM image.

**Fig. 4:**
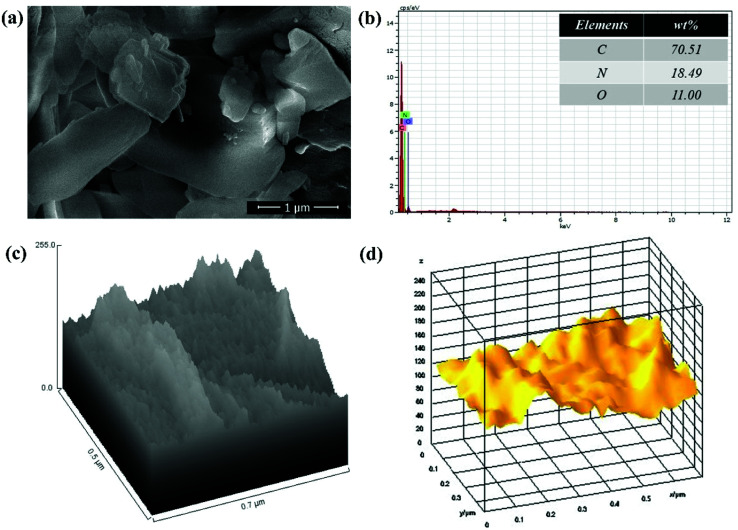
(a) SEM image of GO–PVP, (b) EDX spectra of GO–PVP, (c) Hill stack plot of GO–PVP and (d) 3D surface morphology of GO–PVP.

Also, SEM image of polymer grafted GO in Fig. 4(a) suggested that the polymer is very well grafted over the GO sheets which increase the thickness of sheets. The EDX spectrum of polymer grafted GO in Fig. 4(b) shows the major constituent elements such as C, N and O in 70.51%, 18.49% and 11.00%, respectively, which confirmed the successful functionalization GO with PVP. The Fig. 4(c) and (d) shows the hill stack plot and 3D surface morphology of GO–PVP, respectively. The hill stack plot shows the higher thickness and wider spike like structures in compared to GO, confirm the successful grafting of polymer over the GO surface. Further, same kind of conclusion can be drawn with 3D surface morphology of GO–PVP which confirm the wider spike and thicker sheet like structure.

**Fig. 5:**
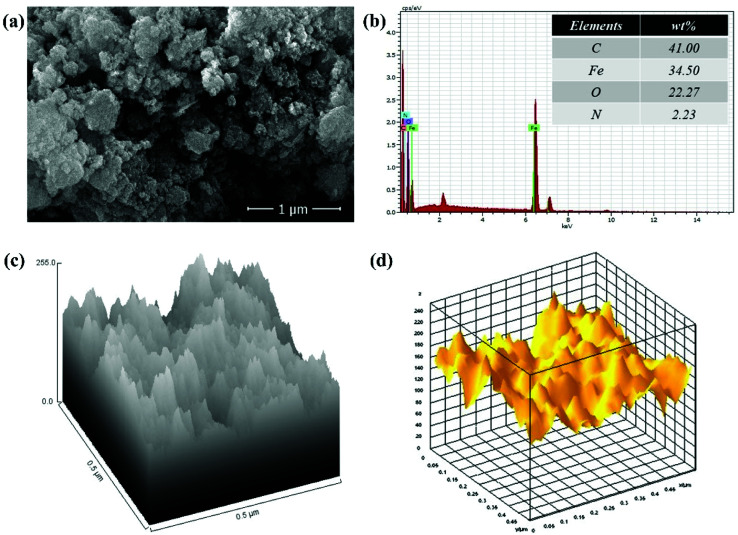
(a) SEM image of GO–PVP–Fe_3_O_4_, (b) EDX spectra of GO–PVP–Fe_3_O_4_ (c) Hill stack plot of GO–PVP–Fe_3_O_4_ and (d) 3D surface morphology of GO–PVP–Fe_3_O_4_.

The SEM image of GO–PVP–Fe_3_O_4_ nanocomposite in Fig. 5(a) exhibited a rough surface along with a good distribution of Fe_3_O_4_ magnetic nanoparticles onto polymer grafted GO sheets. The successful attachment Fe_3_O_4_ magnetic nanoparticles on GO sheet was confirmed by the EDX analysis in Fig. 5(b) which indicated the presence of element Fe (34.50%) along with other constituent elements such as C, O and N in 41.00%, 22.27% and 2.23%, respectively. The Fig. 5(c) and (d) represent the hill stack view and 3D surface morphology of GO–PVP–Fe_3_O_4_, respectively. These figures also show external surface morphology of GO–PVP–Fe_3_O_4_. The hill stack plot indicates the somewhat spike like structure and roughness of GO–PVP–Fe_3_O_4_. This type of structure also confirmed by the 3D surface morphology of GO–PVP–Fe_3_O_4_ in which broader hump like assembly are observed.

### 4.4 Thermogravimetric analysis (TGA)

**Fig. 6:**
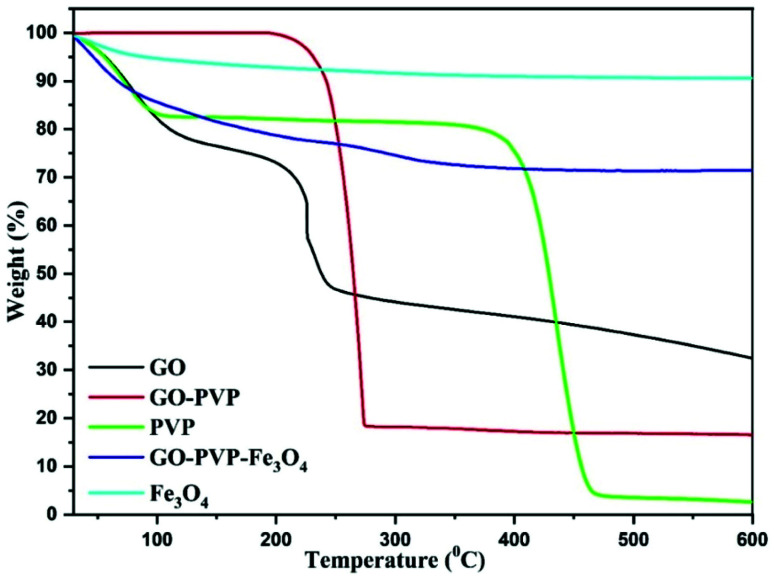
TGA curves of GO, GO–PVP, PVP, GO–PVP–Fe_3_O_4_ and Fe_3_O_4_.

TGA is a characterizing technique for determining the quantity and change in a mass of a material as a function of temperature or time under controlled atmosphere.^41^ TGA thermograms of synthesized materials were recorded in an inert nitrogen atmosphere and the results are shown in Fig. 6. From TGA spectra of GO, it is clearly observed that GO is thermally not stable and shows three stages of weight losses. The initial weight loss was observed at temperatures below 100 °C, which is due to the evaporation of adsorbed water molecules in π-stacked structure of GO. The second weight loss was occurred around 190 °C due to decomposition of labile oxidative functional groups. Finally, the third gradual weight loss was observed in the range of 250–600 °C which is attributed to the ignition of carbon framework. The TGA curve of GO–PVP clearly shows that the thermal stability of GO was significantly improved after the functionalization with PVP. At 600 °C, the TGA data for GO–PVP showed 83.45% weight loss, whereas GO and pure PVP showed weight losses of 67.56% and 97.41%, respectively. As a result, we can estimate that the GO–PVP composite has around 46.77% GO and 53.23% PVP. The TGA curve of Fe_3_O_4_ and GO–PVP–Fe_3_O_4_ showed about 9.44% and 28.53% weight loss over the temperature ranging from 30 to 600 °C. The above TGA data revealed that the GO–PVP–Fe_3_O_4_ composite has about 25.79% GO–PVP and 74.21% Fe_3_O_4_. Therefore, the synthesized nanocarrier GO–PVP–Fe_3_O_4_ showed excellent thermal stability due to the inorganic nature of Fe_3_O_4_ nanoparticles in the nanocomposite.

### 4.5 HR-TEM analysis

**Fig. 7:**
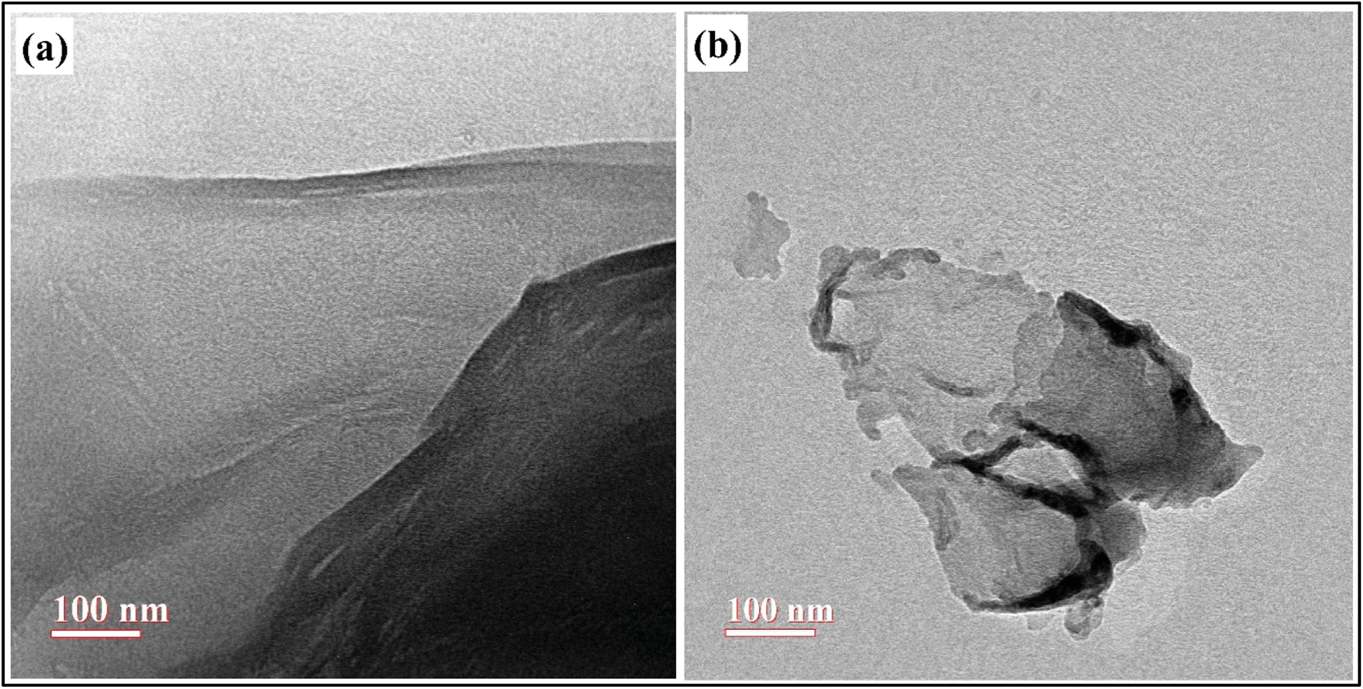
TEM images of (a) GO and (b) GO–PVP.

The morphologies of as-prepared nanocomposites as well as the surface functionalization of GO with PVP was further investigated by HR-TEM analysis and the results are shown in Fig. 7. The wrinkled sheet like morphology of GO was clearly shown in Fig. 7(a), reflecting the successful synthesis of GO by the exfoliation of graphite powder using modified Hummer's method.^21^ The Fig. 7(b) shows the TEM image of GO–PVP, which suggested that the surface of GO has been covered by the polymer PVP and thus, the edges of GO–PVP become relatively coarse.^17^ Also, the thickness of GO sheets was increased after the functionalization with PVP, as seen in Fig. 7(b).

### 4.6 Drug loading

**Fig. 8:**
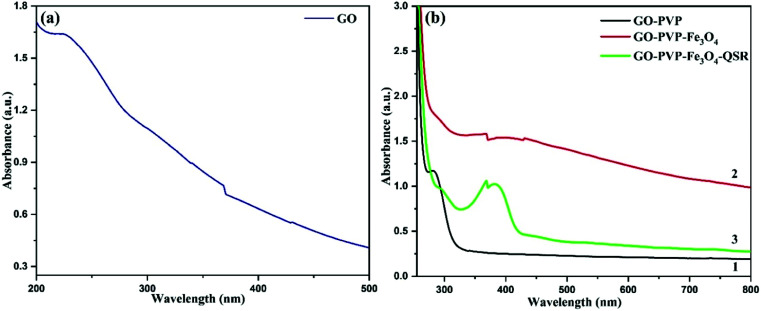
UV-vis spectra of (a) GO, (b-1) GO–PVP, (b-2) GO–PVP–Fe_3_O_4_ and (b-3) GO–PVP–Fe_3_O_4_–QSR.

The loaded amount of drug as well as the entrapment efficiency of drug onto the synthesized nanocarrier are determined by using UV-vis spectroscopy. Fig. 8(a) and (b) shows the UV-vis spectra of GO, GO–PVP, GO–PVP–Fe_3_O_4_ and GO–PVP–Fe_3_O_4_–QSR. A band appeared at 225 nm in Fig. 8(a) due to the π–π* transitions of the aromatic C=C bond which is a significant peak for GO. In Fig. 8(b-1), a sharp rise around 280 nm confirms the functionalization of GO with PVP. In Fig. 8(b-2), the shift from 280 to 288 nm for GO–PVP attributed to the conjugation of Fe_3_O_4_ on the surface of GO–PVP. Also, a broad absorption band around 330–470 nm which confirms the presence of Fe_3_O_4_ in the synthesized nanocarrier. The absorption band appeared at 330 nm confirms the synthesis of nanosized Fe_3_O_4_ particles.^42^ The sharp UV-vis peak at around ∼380 nm assigned to the QSR molecule which was identified in the UV-vis spectra of GO–PVP–Fe_3_O_4_–QSR, as shown in Fig. 8(b-3) which confirms the successful loading of QSR onto the GO–PVP–Fe_3_O_4_.

QSR is a hydrophobic anticancer drug, which commonly used for chemotherapy and was selected for studying the ability of GO–PVP–Fe_3_O_4_ as an anticancer drug carrier. QSR was loaded over the GO–PVP–Fe_3_O_4_ surface by simple mixing and sonication methods through hydrophobic interactions and π–π stacking between QSR and GO–PVP–Fe_3_O_4_. The QSR loading capacity of GO–PVP–Fe_3_O_4_ hybrid was determined by UV-vis spectrum at 380 nm, which was evaluated by the difference between the concentration of QSR in original solution (before loading) and supernatant solution (after loading). The QSR loading capacity of GO–PVP–Fe_3_O_4_ is as higher as 1.69 mg mg^−1^. Further, the entrapment efficiency was found to be 84.8%. The loading capacity of QSR on GO–PVP is only about 0.14 mg mg^−1^ as previously reported.^21^ On the basis of these results, we can conclude that the grafting of Fe_3_O_4_ on GO–PVP surface can effectively improve the QSR loading capacity of GO–PVP. Therefore, GO–PVP–Fe_3_O_4_ can be consider as the excellent nanocarrier with higher drug loading capacity. Further, our developed nanocarrier exhibit the excellent drug loading capacity in compared to other drug carriers, like as liposomes,^43^ polymer micelles,^44^ hydrogel microparticles,^45^ where the loading capacity showed lower than of 1 mg mg^−1^.^27^

### 4.7 Electrochemical analysis

**Fig. 9:**
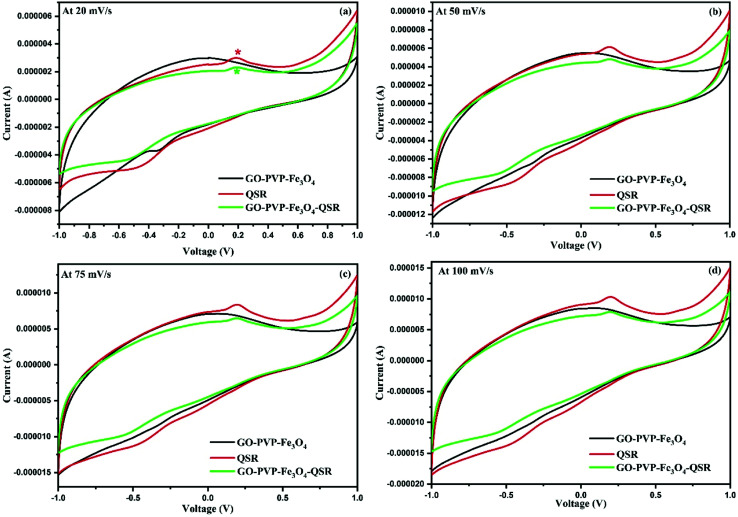
Cyclic voltammogram of the GO–PVP–Fe_3_O_4_, QSR and GO–PVP–Fe_3_O_4_–QSR modified GCE in 0.1 M PBS (pH 7.4) at the scan rates of (a) 20, (b) 50, (c) 75 and (d) 100 mV s^−1^.

Electrochemical analysis was done for identification of attachment of QSR over GO–PVP–Fe_3_O_4_ nanocarrier. For this, we have performed the CV analysis under the voltage range of −1 to 1 V at different scan rates of 20 mV s^−1^, 50 mV s^−1^, 75 mV s^−1^ and 100 mV s^−1^ for GO–PVP–Fe_3_O_4_, QSR and GO–PVP–Fe_3_O_4_–QSR in 0.1 M PBS (pH 7.4) (Fig. 9).

The Fig. 9(a) shows the good cyclic reversibility for GO–PVP–Fe_3_O_4_, QSR and GO–PVP–Fe_3_O_4_–QSR under the performed voltage range. If we compared cyclic behavior of all three, we can see that the GO–PVP–Fe_3_O_4_ does not shows any prominent redox peak (oxidation and reduction peaks) but QSR shows a clear oxidation and reduction peak in the cyclic voltammogram. Further, after loading of QSR over GO–PVP–Fe_3_O_4_ nanocarrier based cyclic voltammogram also shows the prominent oxidation and reduction peaks of QSR at same range. This kind of behavior confirm the loading of QSR over GO–PVP–Fe_3_O_4_ nanocarrier *via* π–π interaction which help to facilitate the electron transport. Such behavior also observed in all cyclic voltammogram at higher scan rates as shown in Fig. 9(b)–(d). All these figures revealed that as we increase the scan rate, reduced the diffusion rate of ions into the electrode and increase ion transport results higher peak current was observed. Therefore, according to the above results we can conclude that the successful attachment of QSR over GO–PVP–Fe_3_O_4_ nanocarrier.

### 4.8 pH-responsive drug release behavior

**Fig. 10:**
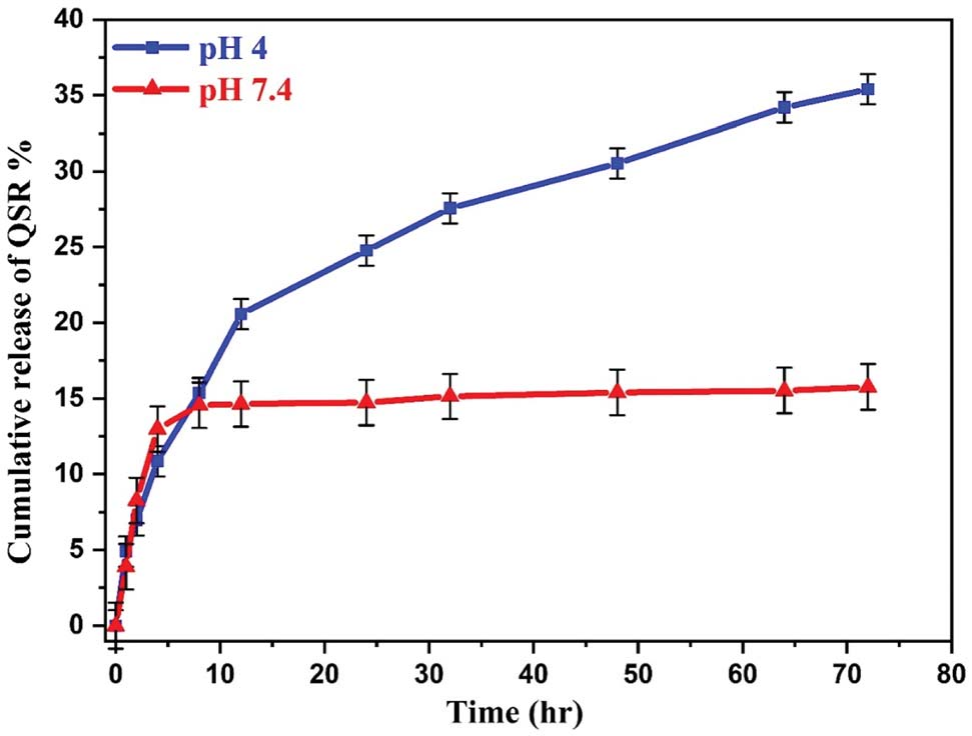
Release profile of QSR from GO–PVP–Fe_3_O_4_ at pH 4 and 7.4.

To evaluate the release performance of the nanocarrier, *in vitro* release study was performed at two different pH conditions, pH 4 and 7.4, which shown in Fig. 10. The release of QSR from GO–PVP–Fe_3_O_4_ nanocarrier was found to be very slow at normal physiological condition and only about 15.76% of the total bound QSR was released for 72 hours under normal physiological condition (pH 7.4). However, in acidic condition (pH 4), the release of QSR was found to be very quick at the early stages and after 12 hours, the rate of release gradually decreases. Finally, 35.40% of the total loaded QSR was released from the nanocarrier in 72 hours at pH 4. This represents the pH-responsive drug release behavior of synthesized nanocarrier.

**Scheme 3:**
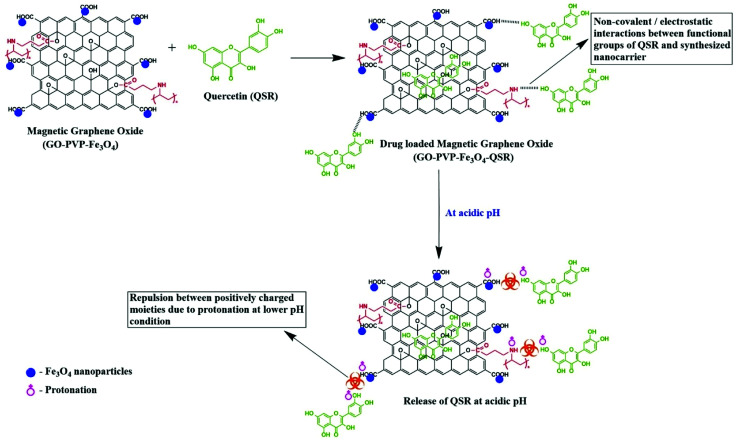
Detailed mechanism of pH responsive release of QSR from synthesized nanocarrier at acidic pH condition.

This may be because of the stronger hydrogen bonding interaction between drug and nanocarrier under normal physiological condition in comparison to the acidic condition, resulting in a slower release rate in normal physiological condition. Generally, tumor cells have acidic lysosomes. As expected for an ideal anticancer drug carrier, the synthesized nanocarrier (GO–PVP–Fe_3_O_4_) initially taken up to the cancer cell *via* endocytosis and finally the drug delivered at cancer cells. So, at lysosomal acidic pH less than 5.5, there is a weakening of hydrogen bonding between QSR and GO–PVP–Fe_3_O_4_ due to the electrostatic repulsion between the positively charged moieties (protonation occur at acidic pH), resulting larger desired release of QSR (Scheme 3). Considering the different releasing behaviors of QSR from GO–PVP–Fe_3_O_4_ at different pH conditions, this synthesized nanocarrier can be consider as a potential carrier for efficient release of drug.^12^

### 4.9 Evaluation of *in vitro* cytotoxicity of QSR loaded nanocarrier against breast cancer cells

**Fig. 11:**
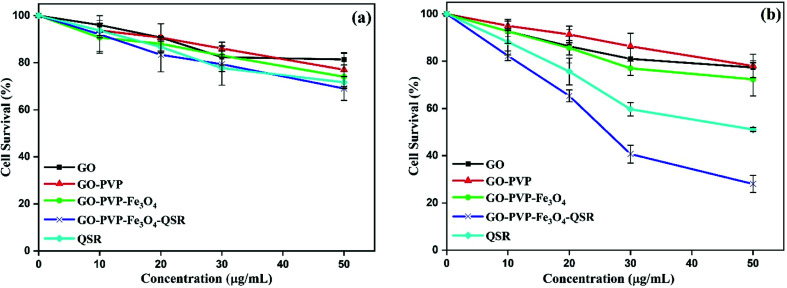
(a) Cell viability of non-tumorigenic HEK 293T cells with various concentrations of GO, GO–PVP, GO–PVP–Fe_3_O_4_, GO–PVP–Fe_3_O_4_–QSR and QSR, and (b) cell viability of human breast cancer MDA MB 231 cells with various concentrations of GO, GO–PVP, GO–PVP–Fe_3_O_4_, GO–PVP–Fe_3_O_4_–QSR and QSR.

The *in vitro* cytotoxicity effect of GO, GO–PVP, GO–PVP–Fe_3_O_4_ and GO–PVP–Fe_3_O_4_–QSR were studied in non-tumorigenic HEK 293T as well as human breast cancer MDA MB 231 cell lines. These cell lines were exposed with different concentrations (0, 10, 20, 30 and 50 μg mL^−1^) of GO, GO–PVP, GO–PVP–Fe_3_O_4_, GO–PVP–Fe_3_O_4_–QSR and QSR. *In vitro* cell viability of both cell lines were shown in Fig. 11. In Fig. 11(a), GO–PVP–Fe_3_O_4_ treated HEK 293T cells reveal no significant cytotoxicity at high concentration of 50 μg mL^−1^. In Fig. 11(b), QSR-loaded GO–PVP–Fe_3_O_4_ shows excellent cytotoxicity against human breast cancer MDA MB 231 cells with around 28% cell viability at 50 μg mL^−1^. Cell survival rate (%) = (*a* − *c*)/(*b* − *c*) × 100 (*a* = absorbance at each concentration of the tested sample, *b* = absorbance at 0 μg mL^−1^ of tested sample, and *c* = absorbance of the blank).

**Fig. 12:**
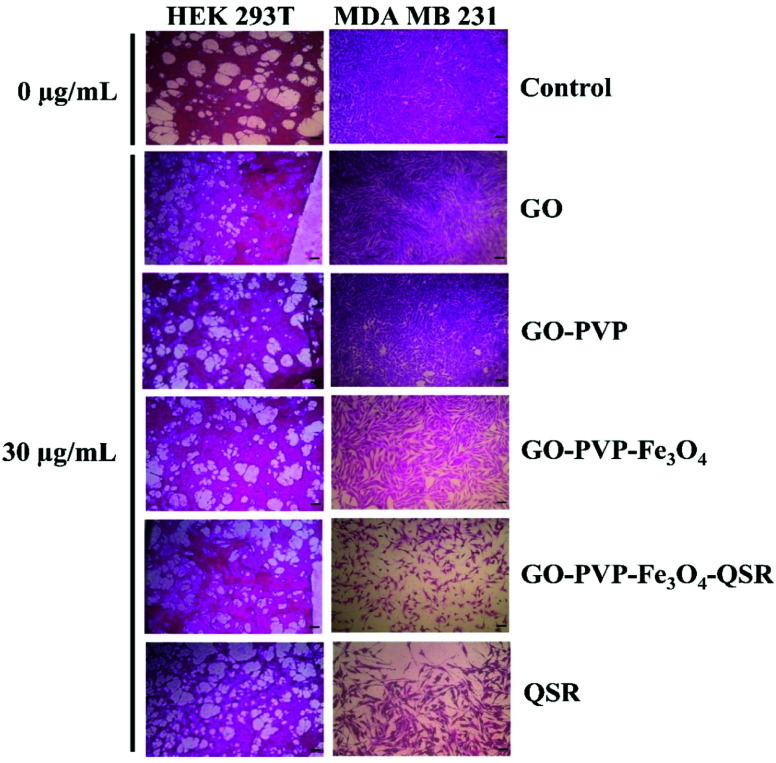
Phase contrast microscopic images of HEK 293T and MDA MB 231 cells after treatment with GO, GO–PVP, GO–PVP–Fe_3_O_4_, GO–PVP–Fe_3_O_4_–QSR and QSR.

In addition, GO–PVP–Fe_3_O_4_–QSR exhibited IC_50_ value in MDA MB 231 cells of 24.20 μg mL^−1^, whereas that of QSR in DMSO is 49.78 μg mL^−1^, suggesting that GO–PVP–Fe_3_O_4_–QSR has significant cytotoxic effects than free QSR against MDA MB 231 cells at the above-mentioned concentrations after treatment of 48 hours. These results indicate that GO–PVP–Fe_3_O_4_ nanocarrier could help to facilitate the delivery of QSR and hence, improve the cellular uptake of the drug. Further, the representative phase contrast microscopic images were captured at various concentrations of synthesized nanocarrier (with and without drug) to investigate the morphological changes of the cells which shown in Fig. 12.

With careful observation, we found that there is no significant cytotoxicity (>80% cell viability) observed for the GO-based nanocarriers at a concentration of 30 μg mL^−1^ for HEK 293T cells (Fig. 12), which indicates the minimal cytotoxicity and favorable biocompatibility of GO-based nanocarriers. However, when MDA MB 231 cells were treated with drug loaded GO-based nanocarriers for 48 hours, a significant decrease in cell viability was observed. Therefore, it is concluded that GO–PVP–Fe_3_O_4_ could be a promising candidate for efficient delivery of QSR with considerable cytotoxicity towards the selected cancer cells.

## 5. Conclusion

In summary, we have successfully synthesized biocompatible and pH responsive magnetic nanocarrier GO–PVP–Fe_3_O_4_ for the efficient delivery of anticancer drug quercetin (QSR). GO–PVP–Fe_3_O_4_ could form stable complexes with QSR through noncovalent interactions and shows high drug loading capacity up to 1.69 mg mg^−1^. We have also shown that in comparison to the free QSR alone, GO–PVP–Fe_3_O_4_–QSR exhibited higher cytotoxic effects to the human breast cancer MDA MB 231 cells at the various concentrations. Overall, our results highlight that polymer grafted magnetic graphene oxide could be a smart pH responsive nanocarrier based drug delivery platform for the efficient and controlled release of anticancer drugs.

## Author contributions

Monika Matiyani: conceptualization, investigation, methodology, experimental and writing – original draft. Anita Rana: analysis and writing, Mintu Pal: experimental, review and editing. Nanda Gopal Sahoo: co-ordinated overall work, review and editing.

## Conflicts of interest

We declare that we have no conflict of interest.

## Supplementary Material
